# Development and Palatability Assessment of Norvir^®^ (Ritonavir) 100 mg Powder for Pediatric Population

**DOI:** 10.3390/ijms20071718

**Published:** 2019-04-06

**Authors:** John B. Morris, David A. Tisi, David Cheng Thiam Tan, Jeffrey H. Worthington

**Affiliations:** 1 AbbVie Inc., North Chicago, IL 60064, USA; cheng.tan@abbvie.com; 2 Senopsys LLC, Woburn, MA 01801, USA; david.tisi@senopsys.com (D.A.T.); jeff.worthington@senopsys.com (J.H.W.)

**Keywords:** Norvir^®^, ritonavir, poorly soluble compound, pediatric, palatability assessment, bioavailability, flavor profile

## Abstract

Norvir^®^ (ritonavir) is a Biopharmaceutical Classification System Class IV compound with poor solubility in water (~5 µg/mL) and limited oral bioavailability. Early stage development efforts were focused on an oral solution (OS) which provided reasonable bioavailability but exhibited taste-masking challenges and required the use of solvents with potential pediatric toxicity. Norvir^®^ oral powder, 100 mg (NOP) was developed to replace OS. The objective of this study is to provide an overview of the development of NOP and palatability assessment strategy. Palatability of NOP was assessed using the flavor profile method: (1) As an aqueous suspension dose/response and (2) evaluation with foods. The dose/response sensory analysis indicated that NOP has strong intensity bitterness and burnt aromatics (3 on the 0–3 flavor profile scale) at the clinical dose (100 mg/10 mL) and the recognition threshold was determined to be 0.3 mg/10 mL. To improve palatability, 100 mg/10 mL NOP aqueous suspension was evaluated with foods. Consuming foods high in fat and/or sugar content after NOP administration successfully reduced bitterness to a 1.5 intensity. In summary, NOP provides dose flexibility, enhanced stability, eliminated solvents, and maintains consistent bioavailability, with reduced bitterness and improved palatability via administration with common food products.

## 1. Introduction

Norvir^®^ (ritonavir) is an inhibitor of human immunodeficiency virus (HIV) protease and is indicated in combination with other antiretroviral (ARV) agents for the treatment of HIV-1 infection [1,2]. Due to the CYP3A inhibitory capabilities of ritonavir, it is also co-administered at lower doses as a pharmacokinetic (PK) enhancer to increase exposures of other HIV protease inhibitors (PIs) [3]. When used as a PK enhancer, ritonavir is most commonly administered at 100 to 200 mg once or twice daily [4]. As a PK enhancer, ritonavir has become a mainstay in the management of both treatment-naïve and treatment-experienced patients and is typically no longer prescribed as a sole protease inhibitor in antiretroviral regimens today [5,6,7]. The PK enhancement often allows for a reduction of pill burden, dosing frequency, and food restrictions, while maintaining efficacy [6,8]. 

Ritonavir is practically insoluble in water (~5 µg/mL), however, this solubility can be enhanced to 1.2 mg/mL at approximately pH 1 (0.1 N hydrochloric acid solution) [9]. Due to the lack of aqueous solubility, ritonavir showed essentially no bioavailability in an animal model (dog) when administered as an unformulated solid in a capsule [9]. Attempts to enhance the oral bioavailability of ritonavir from a solid dosage form by incorporation of surfactants, acids, and other wetting agents failed to increase the bioavailability to greater than ~4%. Similar results were obtained by substituting ritonavir base with salt derivatives of ritonavir. However, bioavailability of 37% in dogs was achieved with a solution formulation containing 5.0 mg ritonavir per mL in a solvent system consisting of 20:30:50 ethanol:propylene glycol:water [2]. Therefore, early formulation development of ritonavir was focused on oral solutions (OS) because of the poor oral bioavailability observed with solid oral dosage forms and the inability to find a solvent system that was compatible with hard or soft gelatin capsules.

OS development efforts concentrated on increasing the drug loading while maintaining oral bioavailability. It was concluded that: a) The relative bioavailability of ritonavir in liquid formulations is inversely proportional to the drug concentration and b) the presence of a surfactant, such as Cremophor EL, enhances the bioavailability of ritonavir in liquid formulations. OS also has a short shelf-life of six months at ambient storage to ensure ritonavir remains in solution (physical stability) while maintaining acceptable levels of degradation (chemical stability).

In addition to bioavailability and stability challenges, OS is known to possess multiple aversive sensory attributes: Basic taste, aromas, trigeminal irritation, and textures, collectively known as “flavor”. These include bitterness and aromatic off-notes from the active pharmaceutical ingredient (API) and trigeminal irritation from the solvents. To address these aversive attributes, an identifying flavor system comprised of sweeteners and aromas (peppermint and caramel) was developed. 

In a preliminary study, the sweetened/flavored formulation somewhat reduced aversive sensory attributes, but overall, the formulation remained relatively low in palatability. Five dose-administration approaches to further ameliorate the sensory effects of OS were subsequently evaluated, first by a trained adult sensory panel using the flavor profile method to identify the most promising one(s). The results are summarized in Table 1. Chasing liquid dose administration with foods was identified as the most promising approach. Six products were selected for confirmation testing with patients based on differences in form, flavor strength, and mastication characteristics: SnackWells^®^ Fudge Brownie, Freshen-Up^®^ Peppermint Gum, Toast Crackers with Peanut Butter, Oats’n Honey Crunch^®^ Granola Bar, Riesen^®^ Chocolate Chews, and Nutella^®^ Spread. Using a 7-point intensity scale, 74 OS patients rated the strength of the “medicine flavor” remaining in the mouth two minutes after taking the OS (mean score 4.95) and chasing with these six foods. All of the food products tested reduced the intensity of the aversive flavor compared to the OS alone, with mean “medicine flavor” ranging from a score of 4.18 to 1.50. An ideal formulation would not require external vehicles, but when faced with significant formulation technical challenges, this chaser approach was effective [10]. 

The development of an amorphous solid dispersion (ASD) formulation for ritonavir was initiated following the introduction of the Kaletra^®^ (lopinavir/ritonavir) tablet in 2005. Experience gained with solid dispersion technology enabled the successful development of the Norvir^®^ 100 mg tablet containing ritonavir ASD which achieved the desired bioavailability and acceptable ambient chemical and physical stability [11]. The Norvir^®^ 100 mg tablet provides significant benefits to patients and physicians, primarily through non-refrigerated storage compared to the OS, offering more robust stability required for storage under global climatic conditions [12]. However, the need for a liquid formulation remains for pediatric patients and adults who are unable to swallow the tablets. To address this gap, AbbVie developed a new powder formulation, Norvir^®^ oral powder, 100 mg (NOP), to provide a more suitable formulation for the pediatric population and with the intent to replace the marketed OS. The objective of this study is to provide an overview of the development and palatability assessment of the age appropriate NOP.

### Development Overview of NOP

The NOP development program was initiated, primarily to mitigate or eliminate the risk of potential toxicities associated with ethanol and propylene glycol solvents in OS, which contains 43.2% (*v/v*) ethanol and 26.0% (*v/v*) propylene glycol [4,13]. Other excipients such as colorants, flavoring agents, and preservatives found in the OS were also removed from NOP. NOP also facilitates dose preparation and administration, aligned with current and future needs for pediatric patients. 

NOP is manufactured by milling the ritonavir ASD extrudate intermediate and filling the resulting powder into sachets. For dose preparation, NOP is suspended in liquid vehicles or sprinkled on soft foods. The dispersion of NOP produces a supersaturated aqueous solution of ritonavir drug substance that maintains the bioavailability achieved with OS [14]. 

Key design targets for the development of the NOP are:

1. Reduction/removal of the ethanol and propylene glycol solvents;
2. Flexibility to accommodate doses for pediatric patients;
3. An acceptable dosage form for pediatric patients or patients who may have difficulty swallowing a tablet;
4. Storage stability to achieve an acceptable commercial shelf life under long term storage conditions;
5. Bioavailability that maintains comparable pharmacokinetic profiles and exposures to the commercial oral solution;
6. Offer opportunities to improve palatability.

Early formulation development efforts evaluated both uncoated and coated powders. To mask the inherent bitter taste of ritonavir, a methacrylic acid–ethyl acrylate copolymer coating (enteric coating, insoluble in acidic media, and soluble above pH 5.5 to allow dissolution in the intestine) was applied to the uncoated powder. Exposure to various pH environments during dose preparation and administration was taken into consideration when assessing impact on the coated powder and potential for drug release and associated aversive attributes as evaluated in healthy adult volunteers. When exposed to various pHs, the uncoated powder provided a more homogeneous suspension compared to the coated powder. This is an important feature to ensure complete dose administration of the amorphous drug suspension for pediatric doses. The uncoated powder formulation achieves the majority of the key design targets, but still had opportunity to improve palatability for dose administration.

The coated and uncoated powders were evaluated for ritonavir pharmacokinetics and palatability as an aqueous suspension administered within approximately 5 min after suspending. In order to evaluate the potential impact of the dissolved aqueous soluble excipients on the ritonavir pharmacokinetics and palatability, uncoated powder was pre-dispersed and held for approximately 30 min prior to administration to allow dissolution of soluble excipients and suspension of the amorphous drug particles prior to administration. The pharmacokinetic results of these three ritonavir regimens (coated, uncoated, and uncoated pre-dispersed powders) showed comparable bioavailability. The bioavailability for all three regimens ranged from 80%–90% relative to OS. The palatability results from this study indicated that no taste-masking benefit was gained from coated powder formulation as compared to the uncoated powder and OS. 

**Figure 1:**
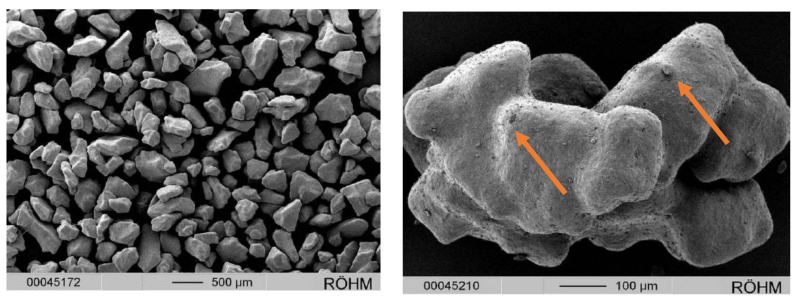
Ritonavir particle shape and small ritonavir particles embedded in eudragit coating. (Orange arrows point to the ritonavir particles).

The initial milled ritonavir ASD extrudate intermediate had a rather broad particle size distribution with a significant fraction of fine particles (<100 µm) and a particle shape that is not ideal for polymeric coating. Coating for conventional tablets, round pellets, or mini-tablets can be effective in reducing or eliminating API taste, resulting in “taste-neutral” formulations [15,16,17]. However, coating for irregularly shaped particulates/granules often results in an imperfect barrier film coat, exposing API in the oral cavity where it can be perceived. The milling and coating processes were not optimized, and it is possible that the finer irregular particles may have had incomplete coating with additional ritonavir particles embedded in the outer polymeric coating layer (Figure 1) leading to potential premature dissolution or exposure to taste receptors during administration. 

**Figure 2:**
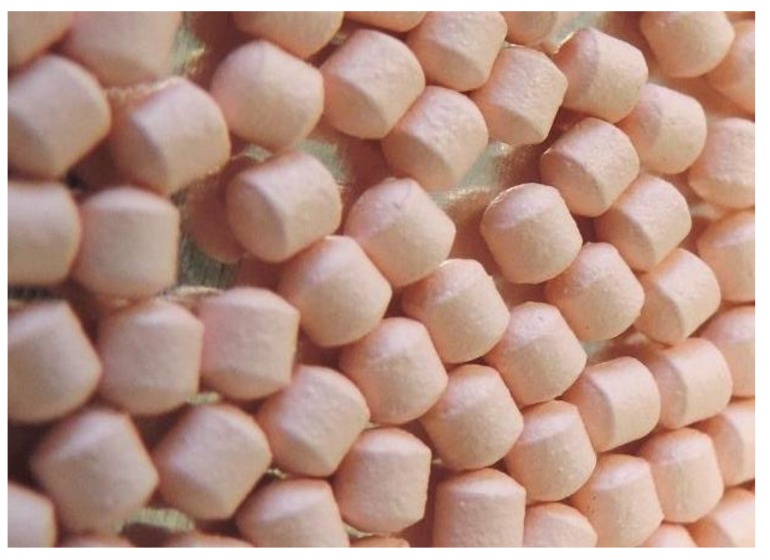
Coated ritonavir extrudate 2-mm particulate.

To further investigate the pharmacokinetic behavior of a larger particle size, still meeting the recommended size for sprinkle products, a uniform 2-mm ritonavir extrudate particulate was coated with the methacrylic acid–ethyl acrylate copolymer (Figure 2) [18]. Although the coated particulate showed improvements in flavor as measured by a trained adult sensory panel, the pharmacokinetic results showed a reduced relative bioavailability of approximately 50% for the coated particulate relative to the OS. The delayed dissolution and drug release profile had a negative impact relative to the known narrow absorption window of ritonavir in the upper intestine. Given the challenges of achieving sufficient coating for taste-masking purposes, without negatively impacting bioavailability, development of NOP was focused on using uncoated powder.

## 2. Results and Discussion

### 2.1. NOP Palatability Assessment, Part 1: Dose/Response Sensory Analysis 

**Figure 3:**
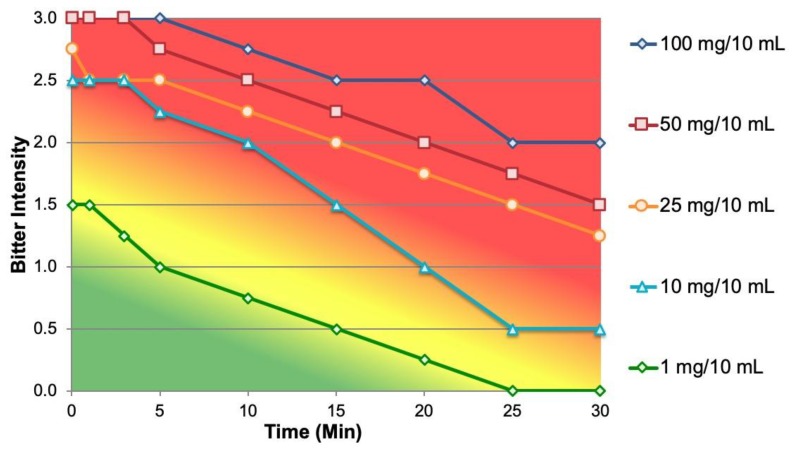
Bitterness dose/response of Norvir^®^ oral powder (NOP).

All five strengths of NOP aqueous suspension were characterized by a strong and lingering bitter basic taste and secondary aromatic off-notes described as “burnt” (polyethylene, wax, and hair). As shown in Figure 3 bitterness of the five strengths spanned the upper half of the flavor profile supra-threshold intensity range, the most challenging from a taste-masking perspective. The bitterness of NOP is very strong at the 100mg clinically relevant dose and all strengths would be patient-perceptible (≥1 intensity) initially and for varying lengths of time in the aftertaste. In the absence of a taste-masking system, it would be necessary to reduce NOP strength to about 0.3 mg/10 mL in order for the bitterness to be imperceptible to patients (i.e., <1 intensity).

**Figure 4:**
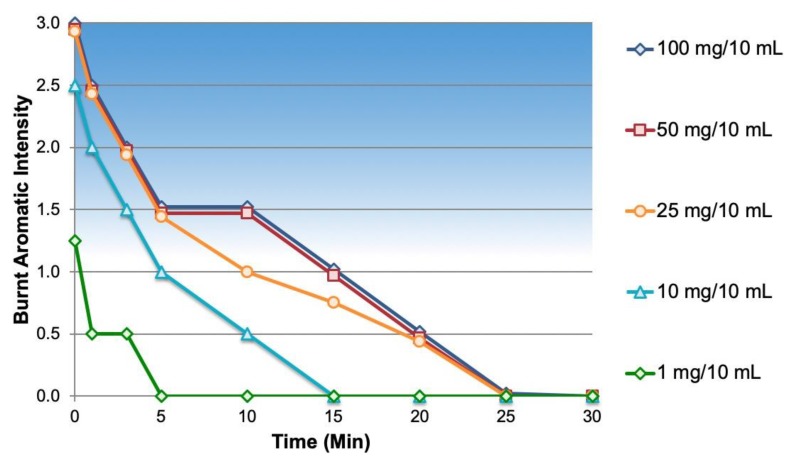
Burnt aromatics dose/response of NOP.

The dose/response results for the burnt aromatics are shown in Figure 4. The four highest strengths of NOP (10, 25, 50, and 100 mg/10 mL) had moderate-to-strong intensity burnt aromatic off-notes. In the absence of an appropriate aroma masking system, it would be necessary to reduce NOP strength to about 1 mg/10 mL for the burnt aromatics to unrecognizable to patients.

### 2.2. NOP Palatability Assessment, Part 2: Evaluation with Foods

**Figure 5:**
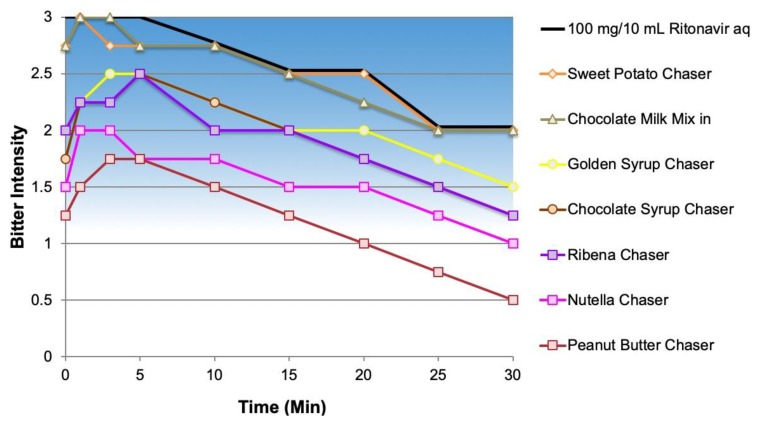
Bitterness profile for NOP with food chasers.

As shown in Figure 5, and consistent with available information, the foods produced varied effects on bitterness reduction, ranging from <½-unit (sweet potato chaser and chocolate milk mix-in) to a 1½-unit reduction in perception of bitterness being associated with food chasers high in fat and/or sugar content with strong flavor intensity (peanut butter, Nutella^®^). The 100-mg clinically relevant dose of NOP is shown for comparison.

**Figure 6:**
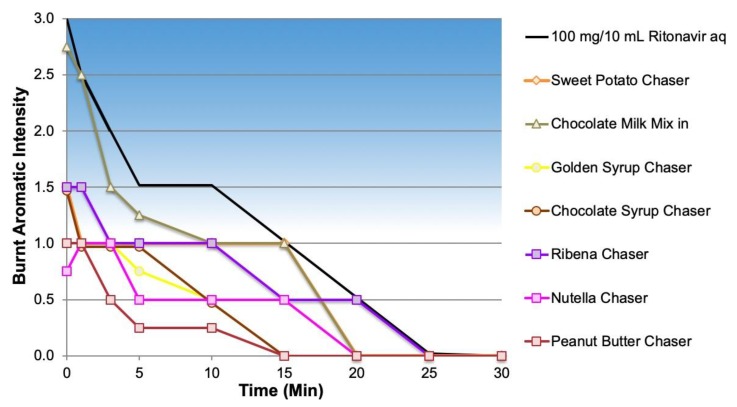
Burnt aromatics profile for NOP with food chasers.

The foods also had varied effects on the burnt aromatic off-notes of the NOP (Figure 6). All of the chasers produced greater reduction in the burnt aromatics than the chocolate milk mix-in (minimal reduction) with several at or below the threshold for perception (≤1). The poor performance of chocolate milk may have been due to its administration as a mix-in, which would have allowed for an extended time for hydration, potentially releasing volatile aromatics.

During the bioavailability studies, palatability of NOP in various beverages (water, infant formula, and chocolate milk) as well as admixed into a soft food (applesauce or vanilla pudding) was compared to that of the OS through study participant questionnaires. Only the NOP mixed in chocolate milk showed a modest improvement in overall palatability compared to the OS. The bioavailability study also demonstrated that administration of NOP with infant formula, chocolate milk, applesauce, or pudding was bioequivalent to administration in water [19]. This observation suggests that NOP may be administered with a wide variety of vehicles (soft foods and liquids) with limited impact on bioavailability.

Based on these results, the foods were ranked in descending order of their overall ability to reduce the aversive sensory attributes of the drug product when administered as chasers. The composition and physical characteristics of foods can be important determinants of their ability to “mask” the aversive sensory attributes of drug products. Table 2 summarizes the fat, sugar content, and water activity of the model foods. Water activity is a measure of relative vapor pressure of water molecules in the headspace above a food versus vapor pressure above pure water from 0 to 1 (pure water). This type of food science-based categorization system will ensure that the most varied food types are tested with a minimum of overlap between samples. The best performing foods may then be screened for chemical compatibility, resulting in specific dosing recommendations that are both effective at reducing the aversive sensory attributes of the drug product and efficacious.

## 3. Materials and Methods

### 3.1. Materials

The NOP was supplied as sachets containing 100 mg milled ritonavir extrudate powder for addition to water for preparation of an aqueous suspension.

### 3.2. Methods

**Figure 7:**
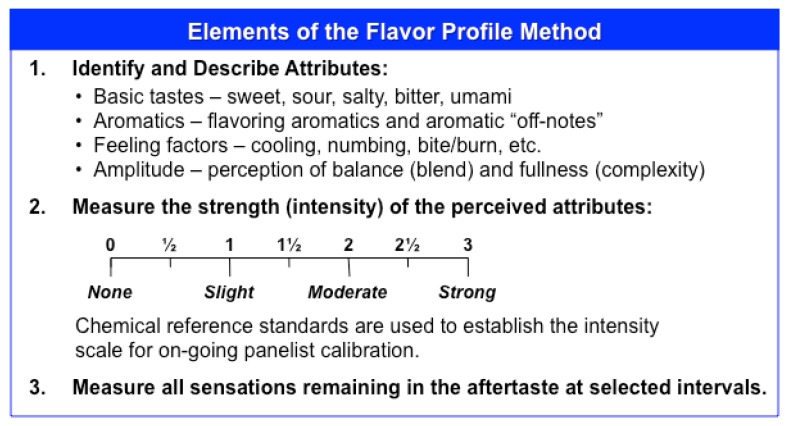
Flavor profile definitions.

Samples were evaluated using the flavor profile method of sensory analysis, an internationally recognized and approved open-source method [20]. Flavor profile is used to identify, characterize, and quantify the sensory attributes of the samples. Flavor profile measures the perceived strength or intensity of the attributes, the order in which they appear; and a description of all flavors—basic tastes, feeling factors, and aromatics—remaining at specified time intervals in the aftertaste. Per the methodology, 4–6 panelists evaluated each sample and collectively arrived at a consensus judgement of the attribute intensity using chemical reference standards to establish the intensity scale. Sensory characteristics above a slight intensity on the flavor profile scale (>1) are clearly perceptible to consumers/patients; this intensity is known as the recognition threshold. Therefore, in order not to be perceived, negative attributes (e.g., bitterness or irritation) should be below this threshold. The flavor profile terms are as shown in Figure 7.

A two-part study was conducted with healthy adult sensory panelists (subjects). Part 1 was a dose/response sensory analysis of NOP to determine the maximum concentration that is patient perceptible. Part 2 was an assessment of the sensory performance of NOP when administered with model foods. The study was conducted in accordance with good clinical practice. 

Part I: Dose/Response Sensory Analysis of NOP

The NOP was prepared by milling ritonavir extrudate intermediate material. To characterize NOP across a range of concentrations, five strengths (1, 10, 25, 50, and 100 mg/10 mL) were evaluated, the highest being clinically relevant. 

A 10-mL aliquot of sample was dispensed into individual 1-ounce plastic cups using a graduated oral syringe and distributed to each panelist. Starting at the same time, each panelist poured the sample into their mouth, swished the contents around the oral cavity for 10 s, and expectorated. The panelists independently evaluated and recorded the sensory characteristics at nine discrete time points (0, 1, 3, 5, 10, 15, 20, 25, and 30 min) using the flavor profile method. Each sample was evaluated twice to generate the final flavor profile. Multiple sessions were required to complete the evaluations to limit exposure to the drug active and minimize sensory fatigue.

Part 2: Evaluation of NOP with Model Foods

There are several approaches that should be considered when improving the “palatability” of drug products with extremely aversive sensory attributes, e.g., bitterness. For NOP, taste-masking was not achievable via a formulation strategy, so two potential alternative approaches were considered, including dosing with food (the term “food” also includes beverages) and cleansing the palate with a food immediately following dose administration (using a food “chaser”) [21]. The principle of food “chasers”, consumption of a food immediately following dose administration, was explored based on historical experience evaluating various approaches with common food products to reduce the bitterness intensity with OS.

The selection of food products is based on multiple factors including patient age, ease of preparation, and availability. Choices for infants and toddlers often include applesauce, yogurt, and formula [22]. However, limited consideration is given to the composition and chemical properties of the foods, e.g., fat, sugar moisture content, pH, water activity, flavor impact (intensity and duration). Seven products were selected in part based on their compositional diversity from a food science perspective as well as availability and fit with the target demographics and geography (e.g., pediatrics in Africa). These products were peanut butter, hazelnut spread, black currant concentrate, golden syrup, chocolate syrup, sweet potato puree, and chocolate milk. Nutrient data was determined from the commercial nutrition facts labels and water activity was measured on an AquaLab Model 4TEV water activity meter.

The commercial food products were evaluated as dosing vehicles for NOP (“mix-in”) or following administration of the NOP (“chaser”) to help cleanse the palate. The sensory panelists first evaluated each of the model food products alone (i.e., without NOP) to develop a flavor profile of the native product.

For dosing with model liquid food (mix-in), the panelists were provided a sachet containing NOP and a 1-oz sample cup containing 10 mL of the model liquid food. Starting at the same time, panelists emptied the sachet in to the model food and mixed. After mixing, panelists took the sample into the mouth and agitated for 10 s and expectorated the liquid. The panelists independently evaluated and recorded the sensory characteristics at nine discrete time points (0, 1, 3, 5, 10, 15, 20, 25, and 30 min) using the flavor profile method. The process was repeated for a second evaluation of each sample to generate the final flavor profile for the sample. Multiple sessions were required to complete the evaluations to limit exposure to the drug active and minimize sensory fatigue.

For evaluating the effects of “chasers”, the panelists were provided a sachet containing NOP, a 1-oz sample cup containing 10 mL water, and a disposable spoon containing 5 g of the tested food chaser. Starting at the same time, panelists emptied the sachet into the water and mixed, forming a suspension. After mixing, starting at the same time, the panelists sipped the NOP suspension into the oral cavity, agitated in the mouth for 10 s, and expectorated the liquid. Immediately following expectoration, panelists took the food chaser from the spoon into the oral cavity, agitated in the mouth for 10 s, and swallowed the food chaser. The panelists independently evaluated and recorded the sensory characteristics at the same nine time intervals using the flavor profile method. As before, the process was repeated for a second evaluation of each sample to generate the final flavor profile for the sample.

## 4. Conclusions

The NOP formulation is an acceptable and age-appropriate dosage form to replace the OS for pediatric patients or patients who may have difficulties in swallowing a tablet. The majority of key design targets were achieved for the development program and suitable palatability assessments (flavor profile method) were executed. The development and taste assessment work conducted for the NOP complements the extensive work previously performed for OS. While it was not feasible to substantially improve the palatability of the formulation itself, there may be widely available options for patients to reduce the lingering bitterness.

NOP may be added to soft food (applesauce, vanilla pudding) or suspended in a liquid (water, infant formula, chocolate milk) as a convenient method for dose administration, though the effects on bitterness reduction were determined in previous studies to be modest. In this study, a variety of beverages and soft foods and consumed immediately after NOP dosing (peanut butter, hazelnut chocolate spread, black currant fruit drink concentrate, golden syrup, and chocolate syrup) were shown to reduce the intensity and duration of the bitter aftertaste, most notably peanut butter and hazelnut chocolate spread. The variety of beverages and soft foods as vehicles, as well as those taken immediately after dosing, offers patients more choices according to their individual taste preferences. 

In summary, the NOP pediatric formulation provides dosing flexibility, enhanced stability and commercial shelf life under long term global climatic (30 °C/75% RH) storage conditions to support use in tropical climates of Africa where more than 95% of children with HIV live, and absence of propylene glycol and ethanol. It maintains consistent bioavailability when administered with a wide variety of vehicles and improved palatability when common food products were employed as “chasers” following dose administration.

## Figures and Tables

**Table 1:** Results of approaches to ameliorate the flavor impact of Norvir^®^ 80 mg/mL oral solution.

Approach	Example Products	Results
Pre-coat mouth to dull sensory receptors	Peppermint Patties (trigeminal cooling); Orange Sherbet (thermal cooling)	No reduction in active pharmaceutical ingredient (API) bitterness or burning mouthfeel.
Mix with food/beverages to dilute sensory effects	Chocolate milk (50/50)	No reduction in API bitterness or burning mouthfeel. Produced a larger volume of an equally bitter solution.
Chase with foods/beverages to wash out aversive sensory attributes	Iced products, milk-based products, fruit juices, savory products, candies, cereals, chewing gums	Solid products reduced the aversive attributes more than liquids. Those more strongly flavored, requiring longer mastication and promoting salivation performed best.
Pre-coat mouth (prime) and chase with foods/beverages	Iced products, milk-based products, fruit juices, savory products, candies	Liquids were ineffective in reducing the aversive attributes. The same solids that performed best as chasers worked as primer/chaser.
Dose with oral syringe	N/A	Produced burning in throat and esophagus and did not reduce bitterness.

**Table 2:** Composition and physical characteristics of model foods selected as dosing vehicle or chaser.

Food	Brand	Quantity	Fat (g/5 g)	Sugar (g/5 g)	Water Activity (a_w_)
Peanut Butter	Jif^TM^ Natural; Creamy	5 g	2.4	0.5	0.251
Hazelnut Spread	Nutella^TM^	5 g	1.5	3.5	0.335
Black Currant Concentrate	Ribena^TM^; Concentrate	5 g	0	2.5	0.912
Golden Syrup	Lyle’s^TM^	5 g	0	5	0.545
Chocolate Syrup	Hershey’s^TM^; Regular Syrup	5 g	0	2.5	0.830
Sweet Potato Puree	Gerber^TM^; 1^st^ Foods	5 g	0	0.2	0.995
Chocolate Milk (Mix-In)	Nesquik^TM^; Lowfat Chocolate Milk	10 mL	0.1	0.6	0.990
